# Eco-friendly nano-enabled fertilizers derived from date industry waste for sustainable and controlled-release of P, K and Mg nutrients: sorption mechanisms, controlled-release performance and kinetics

**DOI:** 10.1186/s40643-023-00716-6

**Published:** 2024-01-03

**Authors:** Samira S. Elsabagh, Elsayed A. Elkhatib, Mohamed Rashad

**Affiliations:** 1 Arid Lands Cultivation Research Institute, City of Scientific Research and Technological Applications, New Borg El-Arab, Alexandria, 21934 Egypt; 2 Department of Soil and Water Sciences, Faculty of Agriculture (El-Shatby), Alexandria University, Alexandria, 21545 Egypt

**Keywords:** Date palm pits, Water retention, Mechanisms of nutrients adsorption, Controlled release, Sustainable agriculture, Nano-carrier, Green byproducts

## Abstract

Development of nano-enabled fertilizers from green waste is one of the effective options to enhance global agricultural productions and minimize environmental pollution. In this study, novel, eco-friendly and cost-effective nano- enabled fertilizers (NEF) were synthesized using the planetary ball milling procedure. The NEF (nDPF1and nDPF2) were prepared by impregnation of nanostructured date palm pits (nDPP) with (KH_2_PO_4_ + MgO) at 1:1 and 3:1 (w/w) ratios respectively. The nDPP, nDPF1 and nDPF2 were extensively characterized. The produced nano-fertilizers enhanced soil water retention capacity with nDPF2 being the most effective. The water retention capacity of nDPF2 treated soil was 5.6 times higher than that of soil treated with conventional fertilizers. In addition, the nDPF2 exhibited superior sustained lower release rates of P, K and Mg nutrients for longer release periods in comparison with the conventional fertilizers. For instance, P cumulative release percentages from conventional fertilizers, nDPF1 and nDPF2 in soil reached 22.41%, 10.82 and 8.9% respectively within 384 h. Findings from FTIR and XPS analyses suggested that hydrogen bonding and ligand exchange were the main interaction mechanisms of PO_4_-K-Mg ions with nDPP surface. The released kinetics data of the NEF revealed that power function was the best suitable model to describe the kinetics of P, K and Mg release data from NEF in water and soil. Pot study ascertained that the nano-enabled fertilizers (nDPF1 and nDPF2) significantly promoted biomass production and nutrient uptake of maize plants as compared to commercial fertilizer treated plants. The present work demonstrated the potential of NEF to increase nutrients uptake efficiency, mitigate moisture retention problem in arid soils and reduce nutrients loss through leaching and safeguard the environment.

**Graphical Abstract:**

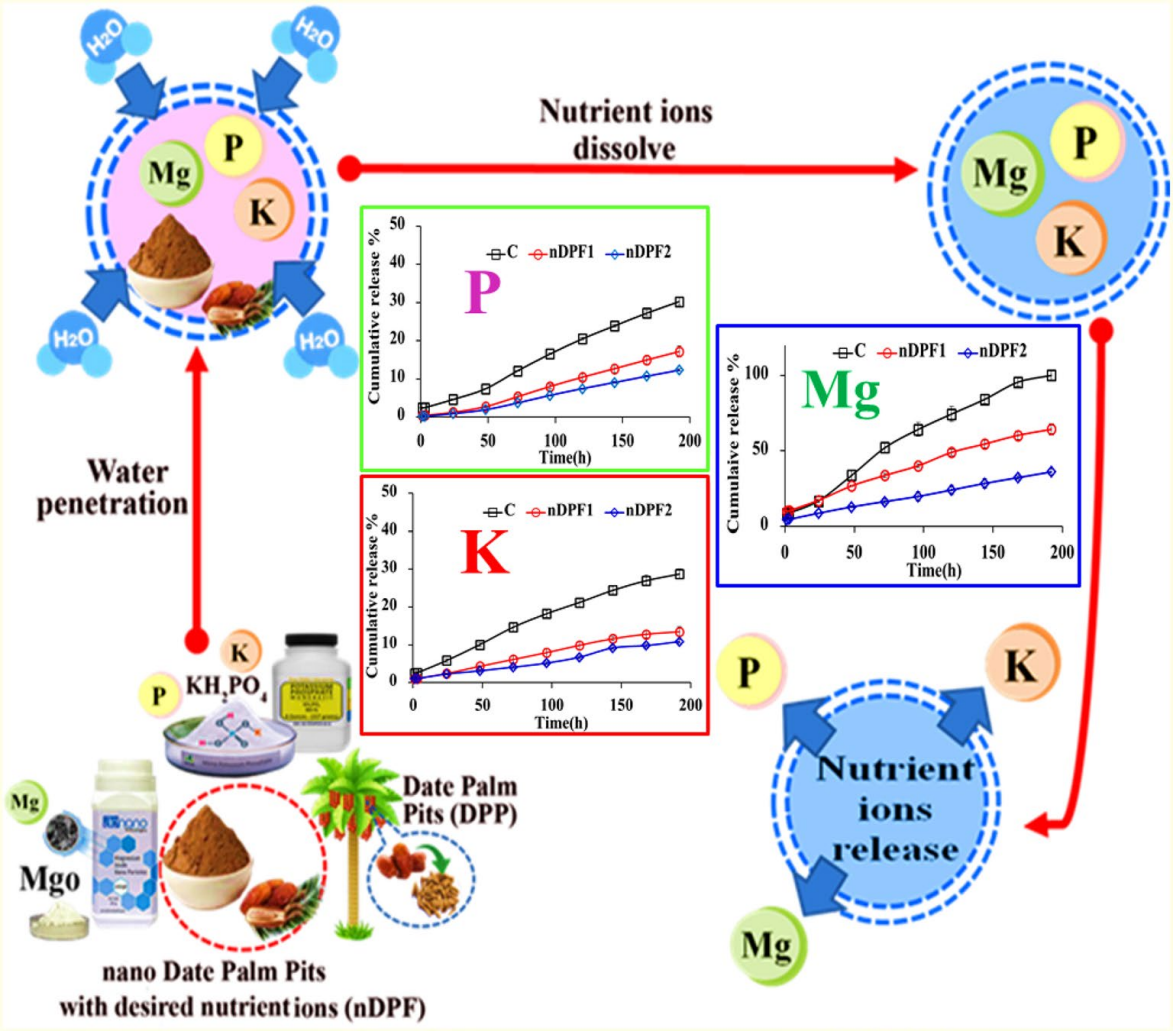

**Supplementary Information:**

The online version contains supplementary material available at 10.1186/s40643-023-00716-6.

## Introduction

Population of the world is predicted to average 9.8 billion in year 2050 (UN.2022), thus a significant demand of world agriculture production is anticipated. FAO (2018) predicted 70% increase in global production of grain by 2050 to cover such demand. To achieve the targeted crop production worldwide in the presence of limited land and water resources, a significant increase in agricultural fertilizer application is required. Meanwhile, exorbitant use of inorganic fertilizers may cause nutrients loss, eutrophication and consequential environmental problems linked to soil and water contamination (Czarnecki and During 2015; Xiao et al. 2019). To manage these economic and environmental obstacles, an indispensable research is needed to develop innovative economic highly efficient fertilizers to improve nutrients supply for optimal plant growth, and to minimize environmental disruptions of globally sustainable agriculture. The choice of using agroindustry wastes for economic gain and the waste utilization could provide food security, environmental safety and sustainability to mankind. These natural materials are low cost, decomposable, save and assist in maintaining soil quality (Cerri et al. 2020; Perez Bravo and François 2020).

Dates fruit and byproducts are widely consumed for their nutritional and curative values worldwide (Al–Farsi and Lee 2008; Sirisena et al. 2015; Djaoudene et al. 2019). Pericarp is an edible part, consumed fresh or in the dried form whereas a pit is considered a byproduct (waste) (Besbes et al. 2004). The date-pits mass represents 10–15% of total mass of date-fruit. The global date production is growing steadily and reached over 1.1 million tons in 2020 and around 125,000 tons of date-pits are produced yearly. Date-pits are generally used as feed additives for poultry and animals, organic fertilizer, oil production for drug and cosmetic industries (Hossain et al. 2014).

The use of innovative nanotechnology in agriculture (i.e., Nano-fertilizer development) is a highly promising approach to significantly control and sustained nutrients release, maximize crop production and minimize nutrient losses into the environment (Azeem et al. 2020; Singh et al. 2016; Fatima et al. 2021).To address these challenges, nano-fertilizers should be supplied to the plants over an extended period of time to noticeably reduce the needed fertilizer application rate. With the aid of cost-effective and eco-friendly ball milling technology, novel economic controlled released nanostructured fertilizers could be developed through incorporating a natural byproduct in nanoscale and macronutrient fertilizers to generate nano-enabled controlled release fertilizers having one or more nanoscale components (Elkhatib et al. 2015; Li et al. 2020; Rudmin et al. 2020). The advantages of using nano-natural carriers are tuning the fertilizers to release nutrients in a controlled manner and providing an agro-environmental solution through the sustainable reuse of natural byproducts sources (Dimkpa and Bindraban 2017; Adisa et al. 2019; Ramli 2019; Liu et al. 2020).

To date, comparatively very few nano-enabled fertilizers (NEFs) have been developed with little is known concerning their potential of agricultural application and safety. Therefore, research is crucially required to explicate ways to enhance resource efficiency through developing economic and natural NEFs. The main goal of this study was to produce nano-enabled fertilizers-based green waste and to evaluate its nutrients release pattern and kinetics. This study is the first to investigate the potential of nanostructured date-pits as a carrier to control the macronutrients release and to provide low-cost and agro-ecological solution through the sustainable reuse of natural sources.

## Materials and methods

### Preparation of date palm pits (DPP)

**Fig. 1:**
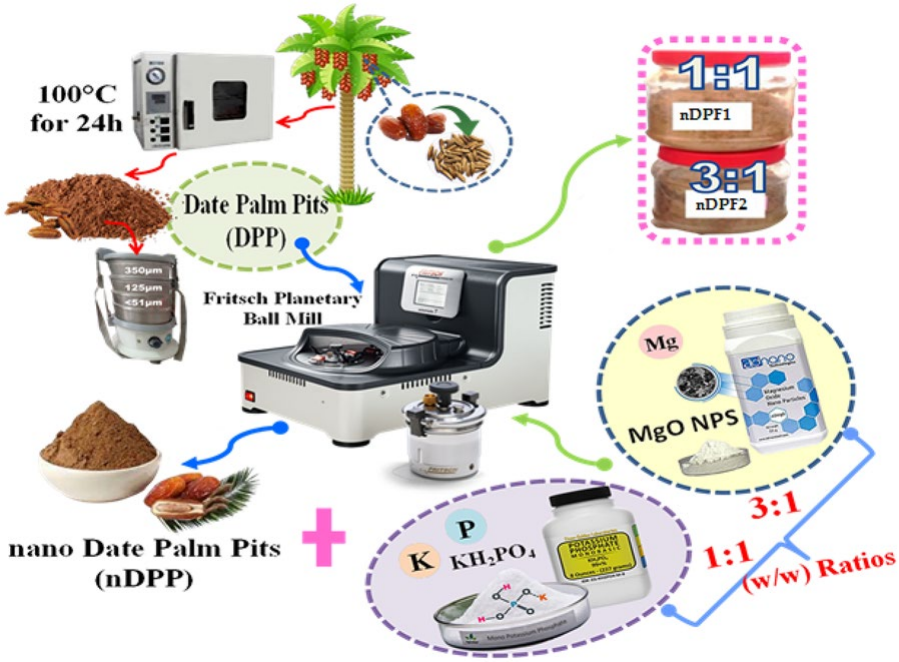
Schematic diagram for preparations steps of enabled nano-fertilizers (ENF)-based date palm pits (DPP)

The date pits (Al-Sultany date) were obtained from the date processing industry (Al-Tahan Dates Company, Alexandria, Egypt) and transported to the soil and water technologies laboratory at Arid Lands Cultivation Research Institute, City of Scientific Research and Technological Applications, New Borg El-Arab, Alexandria, Egypt. The pits were washed in distilled water to remove date flesh and air dried for 3 days and then placed in the oven for 24 h at 100 °C. The dried materials were ground using a stainless steel hammer mill and sieved with 0.51 µm sieve to obtain the date pit powder (Fig. 1). The DPP nanoparticles were produced using a high energy ball mill (Pulverisette-7, Fritsch, Germany) (Elkhatib et al. 2015). The milling process was accomplished by alternating 10 min running and 5 min rest to avoid extravagant heat. The nanostructured DPP (nDPP) carrier was stored in ziploc polyethylene bags until further use. Sizes, shape, surface morphology of nDPP were explored using transmission electron microscopy (TEM) (H-7650, Hitachi, Japan). Crystal structure of nDPP was performed via X-ray diffraction analysis, XRD (Bruker D2 Phaser diffractometer). The diffractogram was recorded in the2θ range = 0–100°. The surface chemistry analysis of the samples produced was performed by X-ray photoelectron spectroscopy (XPS).

### Synthesis and characterization of Nan- enabled fertilizer-based nDPP

The conventional fertilizers (KH_2_PO_4_ and MgO, powder) used were of analytical grade and were used as received without any further purification and were obtained from Sigma–Aldrich. The nanostructured DPP (nDPP) carrier and (KH_2_PO_4_ + MgO) as P–K–Mg nutrients sources at rates of 118 mg P/kg, 150 mg K/kg and 48 mg Mg/kg were mixed at **1 (**nDPP)**: 1**(KH_2_PO_4_- MgO) and **3 (**nDPP)**:** 1 (KH_2_PO_4_-MgO) (**w/w**) ratios and placed in a planetary ball mill (Pulverisette-7, Fritsch, Germany) at 200 rpm for 30 min. Percentages of P, K and Mg in the nutrients sources used (KH_2_PO_4_- MgO) were 22.76%, 28.73% and 60.29%, respectively The produced two impregnated nano-enabled fertilizers are referred to nDPF1 and nDPF2, respectively (Fig. 1).

Scanning electron microscopy (SEM) equipped with energy dispersive X-ray (EDX)analyzer (SEM–EDX, INCAx-Sight model 6587, Oxford instruments, UK) was employed to identify the elemental composition and surface characteristics of the nano-carrier (nDPP) and the produced nano- enabled fertilizers (nDPF1 and nDPF2). The functional groups analysis of nDPP, nDPF1 and nDPF2 was performed by Fourier transform infrared spectroscopy (FTIR, Alpha, Bruker, Germany).

### Soil collection and analysis

A sandy soil (Typic torripsamment) was sampled from EL- Alamien, Alexandria, Egypt at sampling depth 0–15 cm. The collected soil samples were air-dried, ground, and passed through 2 mm sieve. Soil physical and chemical characteristics were determined using standard methodology (Sparks et al. 2001). The measured soil properties are presented in supplemental materials (Additional file 1: Table S1).

### Water retention behavior of nDPF1 and nDPF2 in soil

The water retention behavior of the nano-enabled fertilizers (nDPF1 and nDPF2) in sandy soil were evaluated following the method of Gungula et al. (2021). The detailed description of the method is presented in Additional file 1.

### Controlled release behavior of nDPF1 and nDPF2

The slow release pattern of P, K & Mg nutrients from the fabricated nano-enabled fertilizers (nDPF1 and nDPF2) in distilled water and sandy soils were investigated (Qian et al. 2013; Wei et al. 2019). The experiments are detailed in Additional file 1.

### Controlled release kinetics of P, K and Mg

The P, K and Mg released data obtained from the NEF release experiments were fitted to four different kinetic models (first order, Elovich, Parabolic diffusion and power function) to investigate the controlled release kinetics of P, K and Mg from water and soil amended with NEF and to pin point the best predictive model capable of describing the results (Moharem et al. 2019; Hamadeen and Elkhatib 2022a, b, c).

### Pot experiment

Pilot experiment was conducted to estimate the effect of NEF on maize growth and phosphorus content in plant. Three treatments with three replicates were performed in the pot experiment, comprising the C (control, the soil treated with conventional fertilizers (KH_2_PO_4_ + MgO) at rates of 118 mg P/kg, 150 mg K/kg and 48 mg Mg/kg which are equivalent to 118 kg P/ha, 150 kg K/ha, and 48 kg Mg/ha, respectively), nDPF1 and nDPF2. Percentages of P, K and Mg in the conventional fertilizers used were 22.76%, 28.73% and 60.29% respectively AND all treatments received the same weight of fertilizers. Sandy soil was air-dried, sieved (2 mm) and packed into pots (95 mm diameter, 50 mm deep), with a total of 500 g soil. Firstly, 300 g of the sandy soil was placed in the pots and the fertilizer samples mentioned above were evenly spread on the top of the soil. After that, 200 g of soil was placed to cover the added fertilizer. Then, three seedlings were placed in each pot and the plants were grown for 25 d before harvesting.

All plants were grown under greenhouse conditions and were watered as needed with 50 mL of water (each pot) day after day. At the early seedling stage, the stem diameter and height of the plant were measured. At harvest (25 days), plant shoots and roots were weighed (fresh mass) and dried at 60 °C before being weighed again (dry mass). The tissues were then ground, digested in a 1:3 mixture of nitric acid and hydrochloric acid, and analyzed. The P content in maize seedling was determined using method reported by Reuter and Robinson **(**Reuter and Robinson 1997**).**

### Statistical analysis

All data were analyzed using SPSS (23.0) statistics and Microsoft Excel.

## Results and discussion

### Characterization of nDPP carrier

#### X-ray diffraction (XRD) and transmission electron microscopy (TEM)

**Fig. 2:**
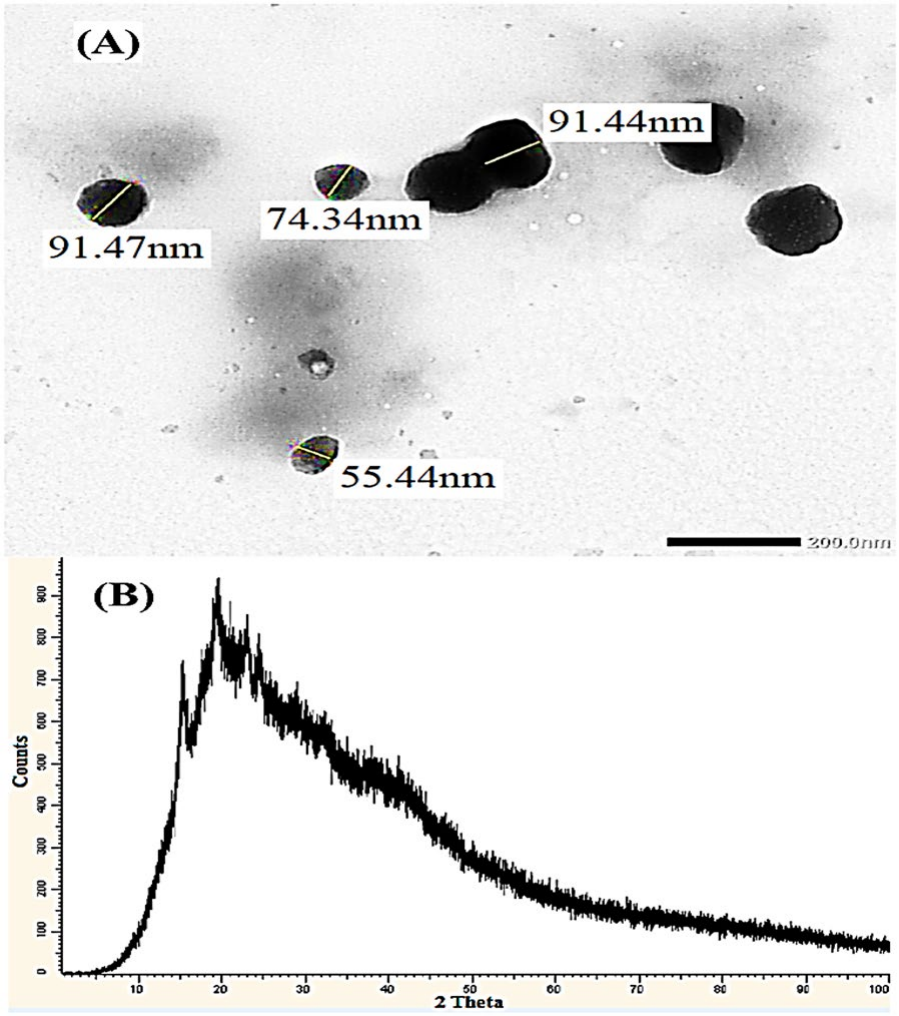
The TEM (**A**) and XRD (**B**) analysis of nDPP carrier

The XRD results of natural byproducts of date industry (date pits) in nanoscale (nDPP) are presented in Fig. 2B. No diffraction peaks analogous to crystallinity were observed, which evidently demonstrated the amorphous nature of DPP nanoparticles carrier. The crystallinity percentage was determined using the empirical method proposed by Wada and Okano (2001). The low value of crystallinity percentage (20.51%) affirmed that the structure of nDPP is composed of a higher number of amorphous domains and suggested the accessibility of nutrients to the nano-carrier*.*

The size and morphology of nDPP were explored using TEM analysis. The TEM image of the nDPP (Fig. 2A) showed that the sizes of DPP nanoparticles range between 55.44 and 91.47 nm and are of non-spherical shape. The irregular and homogeneous structure DPP nanoparticles were in somewhat stable non-aggregated state.

### SEM and EDX analysis

**Fig. 3:**
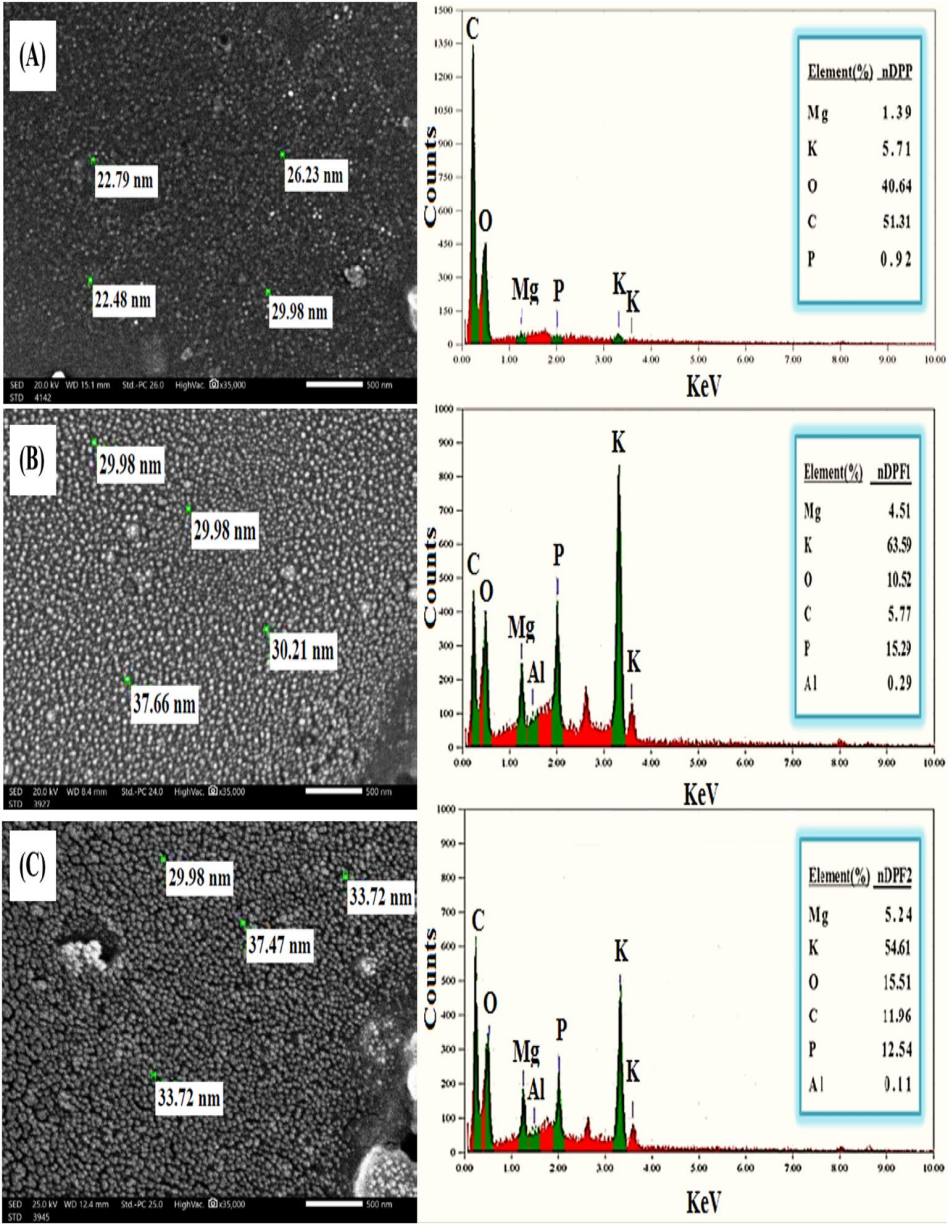
The SEM and EDX images of (**A**) nDPP (**B**) nDPF1 and (**C**) nDPF2

The SEM and EDX were performed to explore surface characteristics, particles arrangement and element compositions of nDPP before and after loading with two different rates of K, P & Mg nutrients. The SEM image of nDPP before loading with nutrients showed irregular structure with different sizes ranging from − 22.48 to − 29.98 nm (Fig. 3A left). The SEM images of nDPF1 and nDPF2 after loading P, K and Mg nutrients are presented in Fig. 3B left and C left respectively. The morphology of nDPF1 and nDPF2 nanoparticles is remarkably different than that of nDPP carrier due to K, P and Mg adsorption processes (Akhtar et al. 2013; Caporale et al. 2013). The SEM images showed clearly coating film of P, K and Mg on the nDPF1 and nDPF2 nanoparticles surface. Meanwhile, the SEM images verified TEM results and affirmed that the particles sizes of the produced fertilizers (nDPF1 and nDPF2) are in the nanoscale.

The EDX elemental analysis of nDPP before P, K and Mg sorption showed high percentages of carbon (51.32%) as well as oxygen (40.65%), and comparably low percentages of K (5.71%), Mg (1.40%) and P (0.93%) (Fig. 3A right). After loading nDPP with KH_2_PO_4_ and MgO, the produced nanostructured fertilizer (nDPF1) showed noticeable increases in potassium (63.59%), phosphorus (15.29%) and magnesium (4.51%) and decreases in C (42.28%) and O (37.71%) contents relative to nDPP (Fig. 3B right). Similar trend is noticed with nDPF2 nanostructured fertilizer due to the impregnation of K, P and Mg nutrients in to nDPP structure (Fig. 3C right). The remarkable changes observed in the SEM and EDX analysis indicate the successful loading and adsorption of P–K–Mg nutrients on the nano-carrier (nDPP) surface.

The zeta potential is important measurable indicator of nanoparticles stability and the degree of repulsion between the charged particles in the dispersion. The zeta potential analysis of nDPP is presented in Additional file 1: Fig. S1A. The negative zeta potential value (− 30.4 mV) of nDPP indicates high negative surface charges and electrostatic stability (Bhagyaraj and Krupa 2020).

### Fourier transmission infrared (FTIR)

**Fig. 4:**
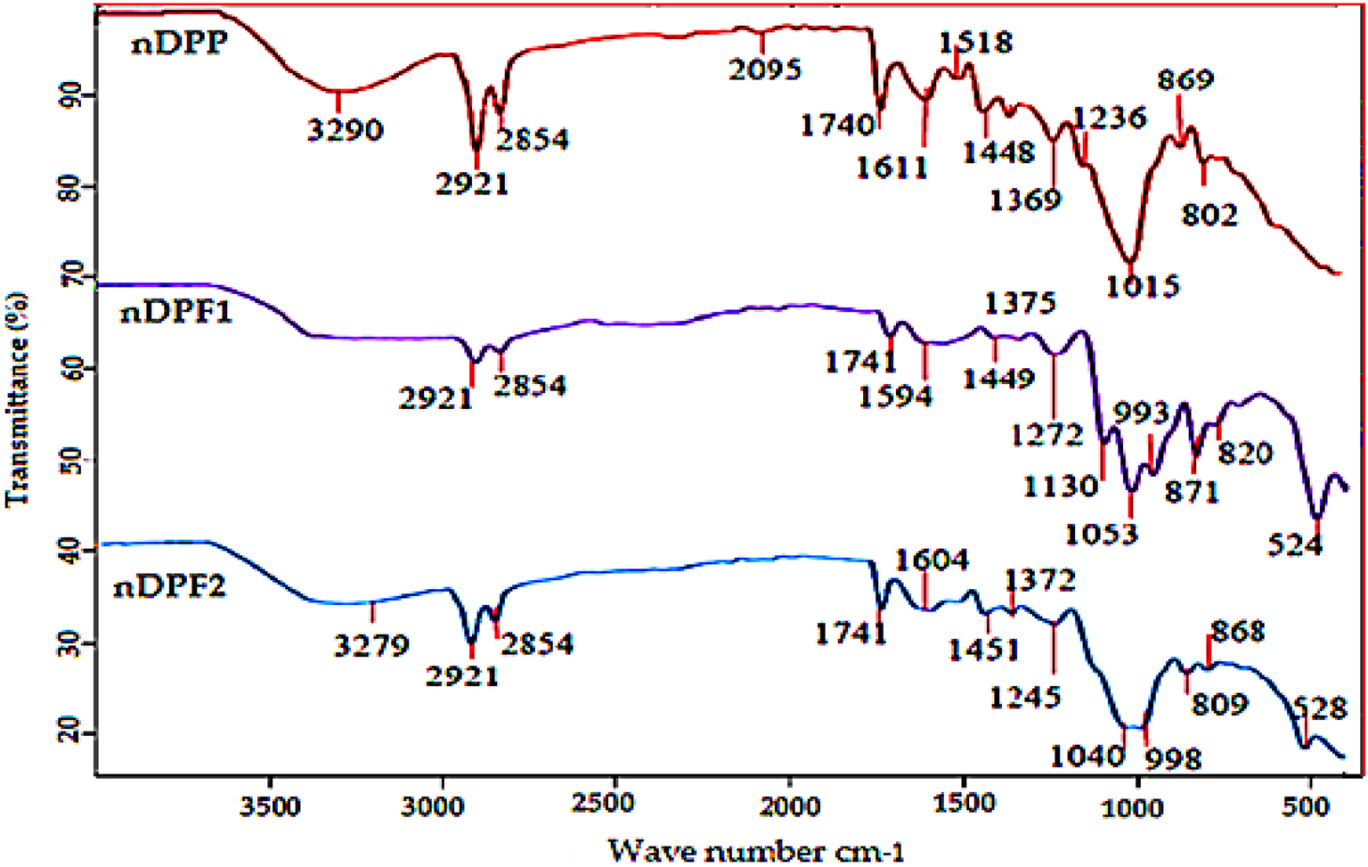
FTIR spectra of nDPP, nDPF1 and nDPF2

The FTIR analysis is a useful tool to recognize the reactive groups on the adsorbent surface and their interaction with the adsorbates. The functional groups on nDPP surfaces and their interaction with KH_2_PO_4_ and MgO addition were explored using FTIR analysis (Fig. 4).The FTIR spectrum of nDPP showed a broad strong band at 3290 cm^−1^ assigned to O–H stretching vibration in association of hydrogen bonding with cellulose, lignin, and pectin(Azam et al. 2021; Pal et al. 2021). Two adjacent bands at 2921 and 2854 cm^−1^ referred to C–H stretching and the carboxylic acid O–H stretch that has been emerged due to aliphatic chains of proteins, carbohydrates and lipids (Mecozzi et al. 2009; Nandiyanto et al. 2019). The band at 2095 cm^−1^ is referred to transition metal carbonyls groups whereas the strong bond at 1740 cm^−1^ is assigned to carbonyl stretch C=O of aliphatic esters which is abundant in hemicellulose (Mecozzi et al. 2009). The bands appeared at 1611 cm^−1^ and 1518 cm^−1^ revealed carbonyl stretch C=C of aliphatic ether and N–O asymmetric stretch of nitro compound, respectively. The two bands emerged at 1448 and 1369 are referred to C-H deformation in lignin and hemicellulose respectively (Al-Ghouti et al. 2010). The band at 1236 cm^−1^ corresponds to C–O stretch in lignin and the band at 1149 cm^−1^ is assigned to C–O–C vibration of alcoholic and carboxylic acids present in cellulose and hemicellulose (Al-Ghouti et al. 2010), whereas stretch band at 1015 cm^−1^ is assigned to silicate ion (Coates 2000). After loading of P, K and Mg onto nDPP, the peaks at 3290, 2095 and 1518 cm^−1^ vanished as is shown in nDPF1 and nDPF2 spectra (Fig. 3). The shift in the nDPF1 and nDPF2 spectral peaks position and appearance at new wave numbers is evident (Fig. 4). The nDPF1 spectral peaks appeared at wave numbers 1741, 1594, 1449, 1375, 1272, 1053 and 820 cm^−1^ whereas the spectral peaks of nDPF2 appeared at new wave numbers 3279, 1741, 1604, 1451, 1372, 1245, 1040 and 809 cm^−1^. The disappearance of C–H band and the changes in strength and spectral peaks position between 3290 and 802 cm^−1^ in nDPF1 and nDPF2 spectra are referred to the interaction of KH_2_PO_4_ and MgO with O–H and C-O bonds of active functional groups present in carbohydrates and proteins (Araújo et al. 2010, 2013). Consequently, disappearance of hydroxyl and carbonyl groups could be due to loading of KH_2_PO_4_ and MgO on nDPF1 and nDPF2 surfaces through adsorption processes.

### X-ray photoelectron spectroscopy (XPS) study of nDPP carrier

**Fig. 5:**
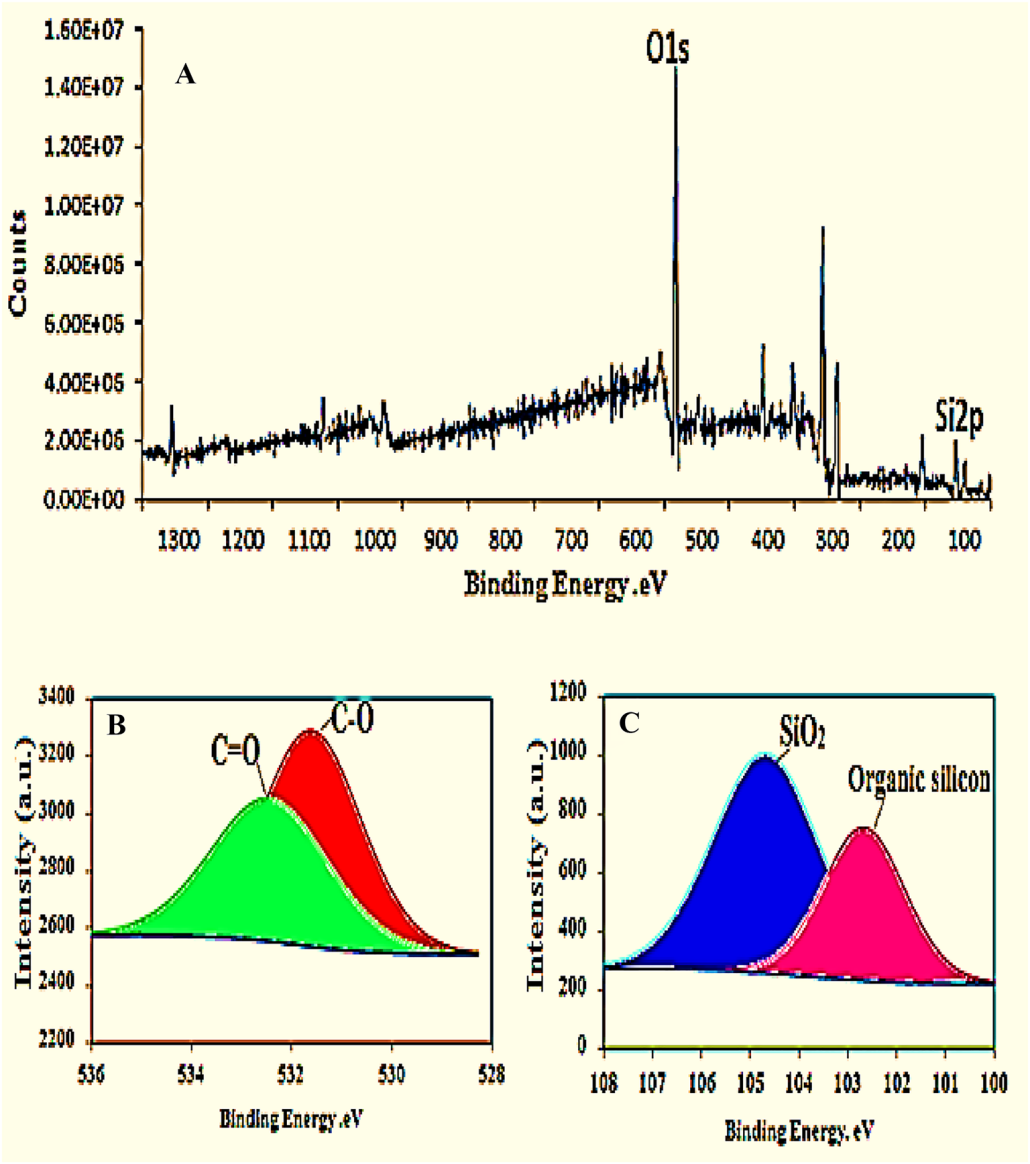
XPS spectrum of nDPP (**A**) full scan spectrum of nDPP shows two major peaks O1s (**B**) and Si2p (**C**)

The XPS study was conducted to further explore the surface chemistry and elemental compositions of nDPP. The full XPS spectra of nDPP display two distinct peaks: the O1s (533.44 eV) and Si2p (105.12 eV) (Fig. 5). The detailed O1s peaks at (531.6 eV) and (532.45 eV) were ascribed to organic C–O and C=O, respectively (Nohira et al. 2002).The Si2p peaks at BE of 104.68eV and 102.67 eV were indicative of SiO_2_ groups and organic silicon respectively (Fig. 5). The XPS assay confirmed that nDPP carrier is mainly composed of C and O, which corresponds with the nDPP elemental EDX analysis (Fig. 3A). Moreover, the O1s, and Si2p spectra of nDPP (Fig. 5B, C) are in compliance with FTIR analysis which identified C=O, C–O and silicate stretching bonds in nDPP sample. The normalized integral areas of peaks and chemical bonding percentages of O1s and Si2 p (Additional file 1: Table S2) indicated that the C=O and C–O groups acted as the active sites for KH_2_PO_4_ and MgO interaction with nDPP.

### The suggested mechanisms for P–K–Mg adsorption by nDPP carrier

**Fig. 6:**
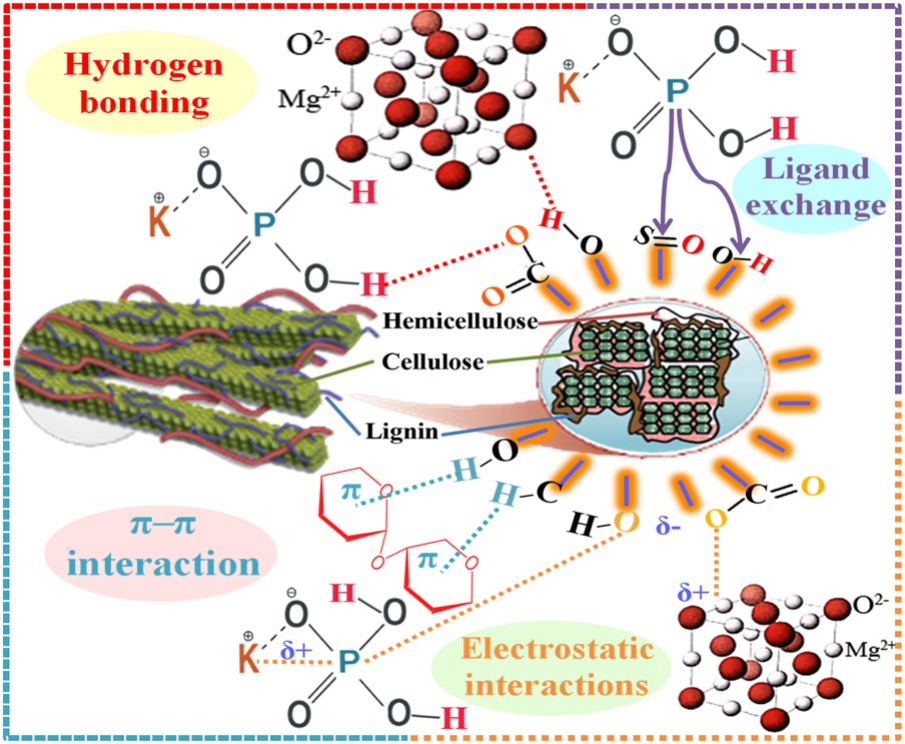
Plausible mechanisms of P–K–Mg adsorption by nDPP carrier

The FTIR spectroscopy technique was used to study the interaction between adsorbates and functional groups on the adsorbent surface. The FTIR interpretation is founded on chemical structure of nDPP carrier before and after loading with K–P–Mg nutrients. Vanishes or shifts of peaks in FTIR spectra demonstrate interactions of functional groups on adsorbents surface with adsorbates. The nDPP is a lignocellulosic material mainly composed of cellulose, hemicellulose, and lignin. The FTIR spectra of nDPP and nDPP- K–P–Mg loaded are shown in Fig. 4. The presence of SiO_2_, C–H, –OH, C–O and C=O functional groups identified in FTIR spectra and XPS analyses indicate their involvement in binding K–P–Mg nutrients by nDPP surfaces. The suggested mechanisms of PO4-K-Mg adsorption onto nDPP carrier are exhibited in Fig. 6. The proposed scheme reveals that the anticipated adsorption mechanisms of PO4-K-Mg nutrients by nDPP are: (1) hydrogen bonding (2) ligand exchange (3) electrostatic interactions (4) π–π interaction.

1. *Hydrogen bonding* The KH_2_PO_4_ & MgO encompass strongly electronegative hydrogen donor (O) whereas the produced nDPP carrier contains hydrogen acceptors (–OH, and C=O). Therefore, it is likely that hydrogen bonding can be formed during the KH_2_PO_4_ & MgO loading process (Fig. 6).
2. *Ligand exchange* The ligand exchange between potassium hydrogen phosphates and sulfonyl functional groups on the surface of nDPP (hemicelluloses) could be one of the processes participating in adsorption of P and K. Meanwhile, the ionized -OH group of cellulose and lignin are involved in the ligand exchange process in accordance with the proposed mechanisms (Fig. 6).
3. *Electrostatic interactions* The strong affinity of nDPP functional groups (–OH, C–O and C=O) for cationic nutrients species of nDPF1 & nDPF2 (K,Mg) greatly indicates the active engagement of electrostatic interactions in the K and Mg sorption processes.
4. *π–π interaction* Functional groups on nDPP surfaces (OH, C–H, C–O and C=O) may operate as π electron-donor whereas π electron cloud of benzene ring in cellulose, hemicellulose and lignin may perform as electron acceptor (Fig. 6).

### Nano-fertilizer parameters and release studies

#### Water retention (WR) behavior of the nano-enabled fertilizer (NEF) in soil

**Fig. 7:**
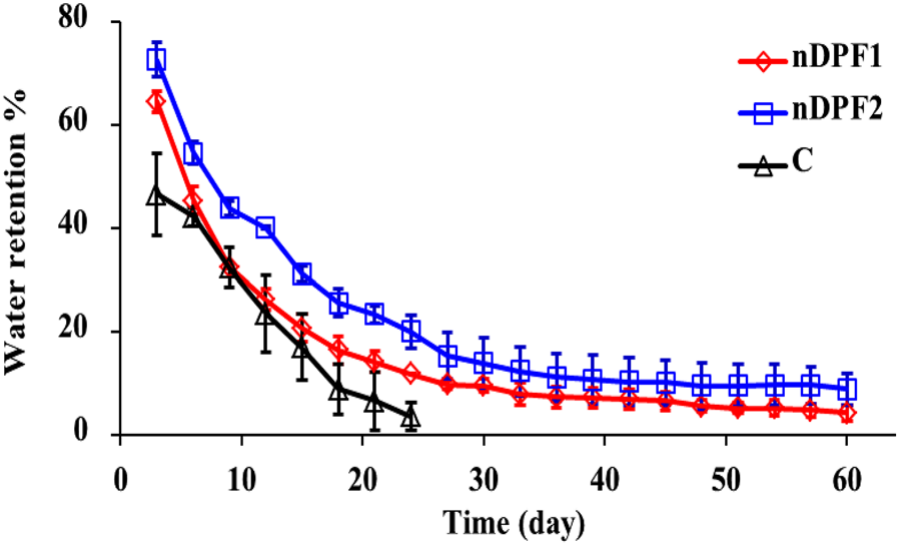
Water retention behavior of soil without and with nano-enabled fertilizers (nDPF1 and nDPF2) Error bars correspond to mean ± standard error of the mean

The nano-enabled fertilizer (NEF) is capable of conserving soil moisture when the material of the nano-carrier is good water absorbent. The water preserved by the carrier during irrigation can be progressively released to the soil to dissolve the mineral nutrients in the nano-carrier for effective release to the plants via diffusion and to fulfil the plant water demands in drought-prone areas (Kong et al. 2019). The WR behavior of soil with and without nano-enabled fertilizers (NEF) was evaluated against time and the gained results (Fig. 7) clearly showed that addition of NEF (nDPF1 or nDPF2) to the soil increased its water retention performance. The WR in the control soil was about 47% on the 5th day, reached 3.6% on the 24th day, whereas, the WR of the soil mixed with nDPF1 and nDPF2 was 64.58% and 72.67% on the 5th day and reached 11.84% and 20.01% on the 24th day, respectively. After 26 days, the water content of the control soil was almost evaporated whereas nDPF1 and nDPF2 fertilizer treatments showed WR values of 4.31% and 8.83% respectively after 60days. It is quite clear that the fabricated nano-enabled fertilizers effectively improved water retention capacity of the soil and slowed down water evaporation with nDPF2 being the most efficient. Therefore, nDPF2 can extend irrigation cycles, and enhance drought tolerance of plants. Similar results have been reported using slow-release urea fertilizer-based starch and hydrogel (Gungula et al. 2021).

### Nutrients release performance of nano-enabled fertilizer (NEF) in water

**Fig. 8:**
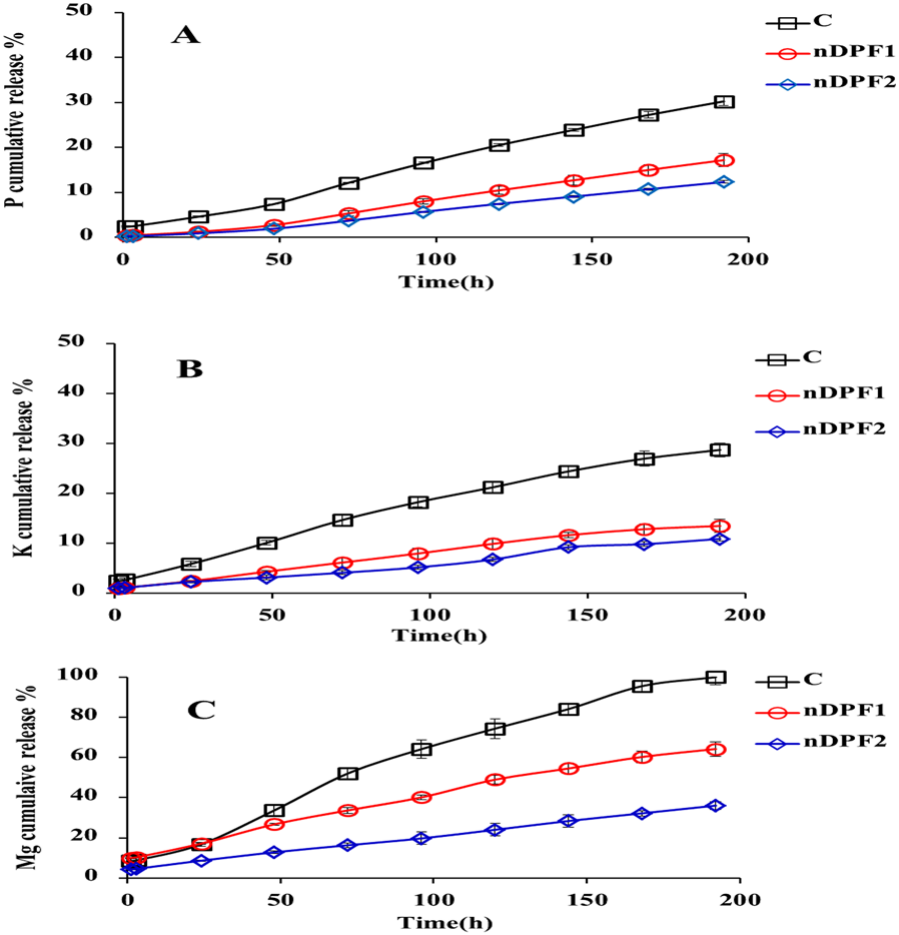
Cumulative release of P (**A**), K (**B**) and Mg (**C**) from control and NEF in water

Nutrient release curve is a key feature characterizing the nutrients controlled release performance of NEF. The release characteristics of P, K and Mg from the fabricated NEF and control were examined in water and the results presented in Fig. 8 indicated that P–K–Mg release rates from control (standard sources, KH_2_PO_4**+**_ MgO) were much faster than P–K–Mg release rates from NEF (nDPF1 and nDPF2). The rate of P released of from control, nDPF1 and nDPF2 in water reached 30.24%, 17.17% and 12.31%, respectively, within 192 h (Fig. 8A). The cumulative release rate of K from control (C) reached 28.74% within 192 h in distilled water (Fig. 8B) as compared to 13.49% and 10.89% of K released from nDPF1 and nDPF2 respectively within the same time frame. For Mg release, the Mg cumulative percentage released from C was about 100% within 192 h in distilled water whereas, the cumulative release percentages from nDPF1 and nDPF2 at the same time interval were 64.25% and 36.05% respectively (Fig. 8C). The P–K–Mg release long duration in tested fertilizers increased with increasing nDPP content. These results demonstrate the potential use of nDPF1 and nDPF2 as promising sustained- controlled release fertilizers with nDPF2 being the most efficient.

### Nutrients release performance of nano-enabled fertilizers (NEF) in soil

**Fig. 9:**
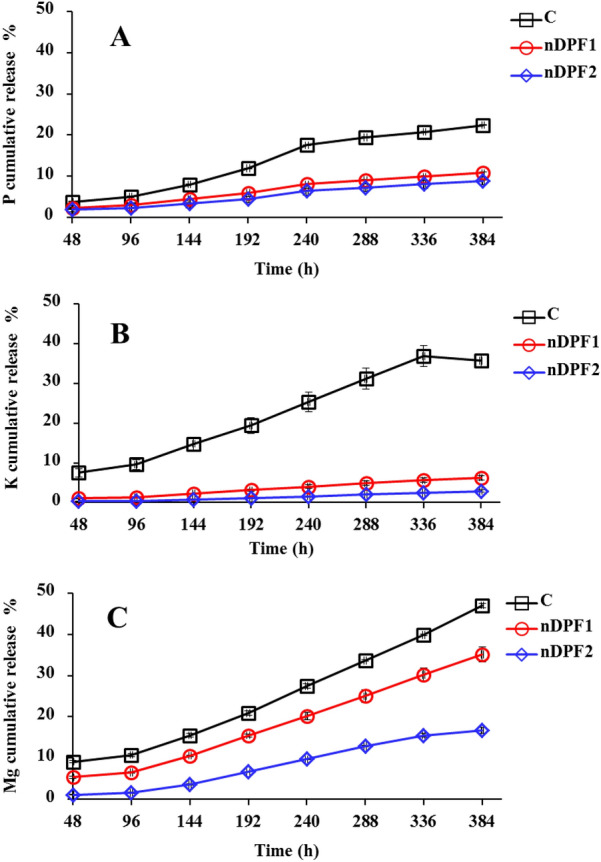
Cumulative release of P (**A**), K (**B**) and Mg (**C**) from control and NEF in soil

The release behaviors of P–K–Mg from classical fertilizer** (**control) and the produced NEF (nDPF1 and nDPF2) were examined in soil column experiment and the results are presented in Fig. 9. The studied nutrients (P–K–Mg) were applied to the soil columns in the form of nDPF1 or DPF2 to improve nutrients availability to plants through alleviating nutrients loss by leaching and enhancing soil nutrient retention thereby reduces fertilizer requirements for optimum plant growth in agricultural soil (Tang et al. 2019). The data presented in Fig. 9 revealed that the rates of P, K and Mg released from control in soil were much lower than those in water Fig. 8. The phosphorus cumulative release percentages from control, nDPF1 and nDPF2 in soil reached 22.41%, 10.82 and 8.9% respectively within 384 h (Fig. 9A). Meanwhile, the cumulative ratio of K released from control was about 35.71% within 16 days (384 h) in soil leachate (Fig. 9B) which is almost 5 and 12 times higher than that of nDPF1 (6.28%) and nDPF2 (2.91%) respectively. Moreover, the Mg cumulative percentage released from control in soil leachate was about 47.01% within 384 h, whereas the cumulative release percentages of Mg from nDPF1 and nDPF2 at the same time interval were 35.13% and 16.71% respectively (Fig. 9C). Remarkably, the P, K and Mg cumulative release percentages in soil leachate were in the following order: Conventional fertilizers > > nDPF1 > nDPF2. It is, therefore, concluded that nDPF2 exhibits a preferable sustained-release property and is considered a promising controlled and sustainable alternative to substitute classical P, K and Mg fertilizers. In addition to the encouraging features of using nDPF2 rather than classical fertilizers to deliver nutrients, the nDPP carrier used is safe, eco-friendly, and adaptable to soil, plants, and other organisms. The excellent P–K–Mg slow-release achievement is accredited to the green carrier characteristics including the high water retention, the high negative surface charges and electrostatic attraction for K and Mg as well as the H-bonds formed during KH_2_PO_4_ & MgO loading process.

### Kinetics and modelling of P, K, and Mg release from nano-enabled fertilizers (NEF) in water and soil

**Fig. 10:**
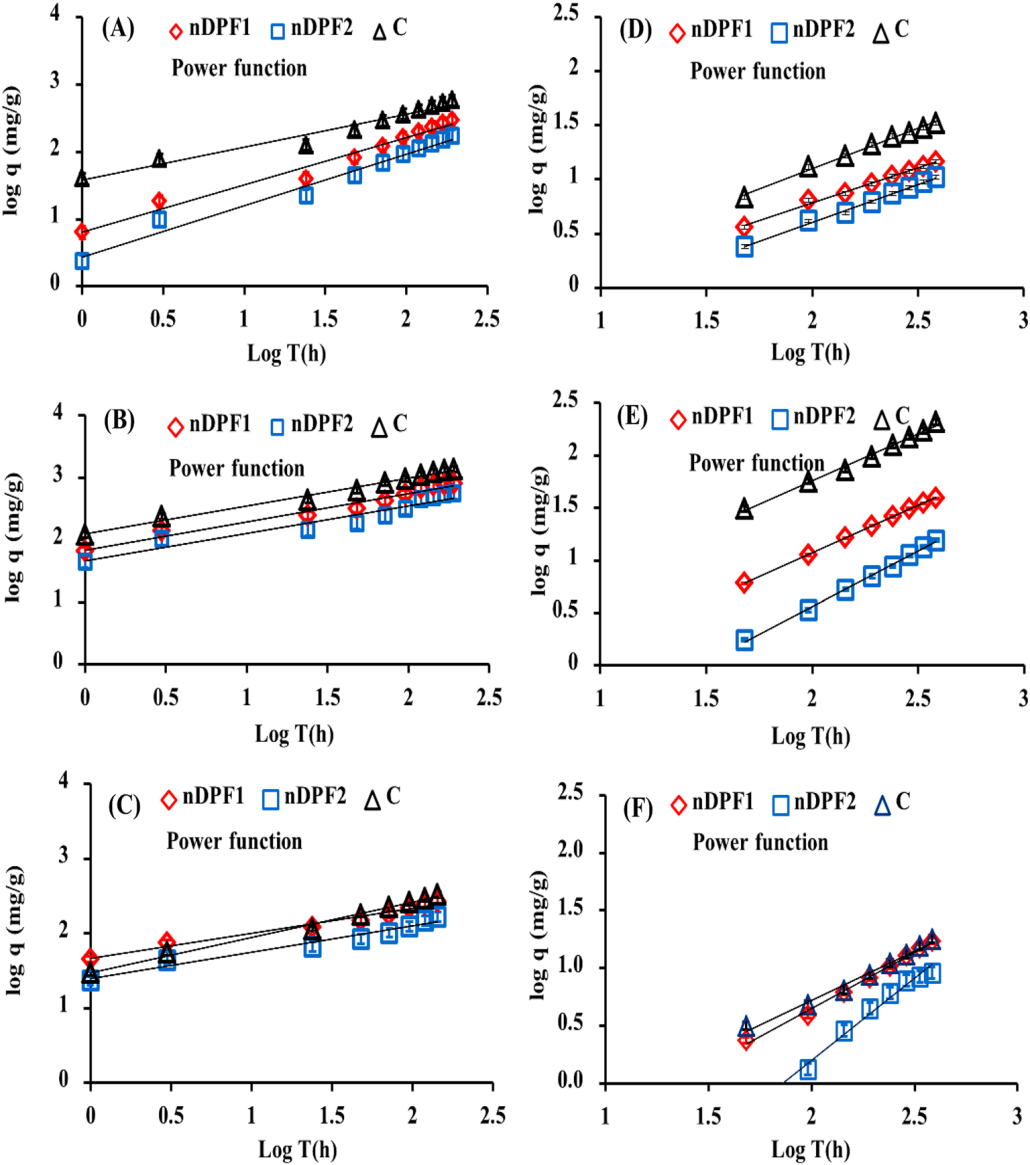
Release kinetic models of P (**A**), K (**B**) and Mg (**C**) in water & P (**D**), K (**E**) and Mg (**F**) in soil

In the present study, the released data obtained from leaching experiments in water and soil were used to assess the suitability of various kinetic models (first order, Elovich, parabolic diffusion, power function and second order) to describe the kinetics of P, K and Mg release from water and soil amended with NEF. The validation of the used kinetic models was based on determination coefficient (R^2^) and standard error of estimate (SE). The five kinetic models tested and its parameters along with R^2^ and SE values are displayed in Tables 1, 2 and 3. The power function model has shown the lowest SE values which indicates that this model is the most suitable model for describing the P, K and Mg kinetics release data from NEF in water and soil (Tables 1, 2, 3 and Fig. 10). In the power function model, the reversibly P, K and Mg adsorbed phases were mainly related to the initial concentrations of P, K & Mg and were proportional to the fractional power of time and controlled by the desorption mechanism (Luo et al. 2021; Hamadeen and Elkhatib 2022a, b, c).

**Table 1:** Kinetics models for P release from classical fertilizers (control) and NEF in water and soil

Models release kinetics	Description	Parameter	Water			Soil		
			nDPF1	nDPF2	C	nDPF1	nDPF2	C
First order Ln (q_0_–q_t_) = a − (K_d_ * T)	q_0_ = amount of P released at equilibrium mg·g^−1^, q or q_t_ = amount of P released at time t (h) mg·g^−1^, K_d_ = Solubility rate (h^−1^) a = constant (mg.g^−1^)	K_d_ a R^2^ SE	0.012 5.835 0.927 0.214	0.012 5.291 0.925 0.215	0.013 6.442 0.942 0.206	0.007 2.835 0.953 0.171	0.006 2.569 0.940 0.191	0.007 3.738 0.967 0.151
Elovich q_t_ = (1/β)ln(αβ) + (1/β)lnt	α = initial desorption rate of P (mg/g·h) β = constant related to P release (mg.g^−1^)	α β R^2^ SE	20.58 0.020 0.742 56.36	11.44 0.034 0.739 32.89	60.59 0.010 0.789 94.78	0.187 0.196 0.961 2.608	1E-01 0.261 0.942 2.417	4E-01 0.080 0.973 1.581
Parabolic diffusion q = a + k_d_t ^1/2^	a = constant (mg.g^−1^) K_d_ = apparent diffusion rate coefficient (mg/g.h^1/2^.)	K_d_ a R^2^ SE	22.44 43.88 0.948 25.27	12.99 26.66 0.946 14.95	42.20 36.07 0.969 36.03	0.840 2.201 0.994 1.009	0.634 2.268 0.989 1.029	2.051 7.405 0.998 1.489
Power function^a^ logq = logk_d_C_ο_ + 1/m logt	K_d_ = apparent desorption rate coefficient(h^−1^) 1/m = constant Co = initial P concentration	K_d_ 1/m R^2^ SE	6.380 0.705 0.978 0.087	2.764 0.765 0.978 0.096	38.17 0.492 0.964 0.078	0.316 0.643 0.993 0.018	0.164 0.696 0.995 0.016	0.410 0.743 0.993 0.021
Second order qt = k_d_q_e_^2^t /1 + k_d_q_e_t	k_d_ = apparent desorption rate coefficient (h^−1^)	K_d_ q_e_ R^2^ SE	0.127 500 0.508 0.149	0.077 344.8 0.451 0.248	0.372 714.3 0.784 0.084	0.017 24.88 0.956 1.094	0.011 20.75 0.912 1.902	0.019 69.93 0.983 0.239

C = Classical fertilizers (control) ^a^SE|≤ 0.1 in all the parameters

**Table 2:** Kinetics models for K release from classical fertilizers (control) and NEF in water and soil

Models release kinetics	Description	Parameter	Water			Soil		
			nDPF1	nDPF2	**C**	nDPF1	nDPF2	**C**
First order Ln(q_0_–q_t_) = a − (K_d_ * T)	q_0_ = amount of K released at equilibrium mg·g^−1^, q or q_t_ = amount of K released at time t (h) mg·g^−1^, K_d_ = Solubility rate (h^−1^) a = constant (mg.g^−1^)	K_d_ a R^2^ SE	0.017 6.805 0.933 0.289	0.016 6.490 0.872 0.386	0.019 7.352 0.908 0.399	0.007 4.031 0.939 0.205	0.006 3.111 0.903 0.220	0.005 5.582 0.942 0.151
Elovich q_t_ = (1/β)ln(αβ) + (1/β)lnt	α = initial desorption rate of K (mg/g·h) β = constant related to K release (mg.g^−1^)	α β R^2^ SE	103.6 0.007 0.818 122.5	65.39 0.011 0.740 102.4	194.1 0.004 0.865 175.4	0.380 0.063 0.940 3.069	1E-01 0.153 0.887 1.770	2E-01 0.012 0.893 22.01
Parabolic diffusion q = a + k_d_t ^1/2^	a = constant (mg.g^−1^) K_d_ = apparent diffusion rate coefficient (mg/g.h^1/2^.)	K_d_ a R^2^ SE	58.82 17.68 0.974 46.76	40.31 26.17 0.934 51.48	98.65 6.99 0.990 46.83	2.652 14.16 0.991 1.206	1.098 7.185 0.962 1.016	13.70 79.07 0.963 12.81
Power function^a^ logq = logk_d_C_ο_ + 1/m logt	K_d_ = apparent desorption rate coefficient (h^−1^) 1/m = constant Co = initial K concentration	K_d_ 1/m R^2^ SE	67.80 0.457 0.973 0.063	46.15 0.444 0.930 0.101	124.2 0.449 0.985 0.046	0.180 0.902 1.000 0.006	0.288 1.054 0.998 0.016	0.900 0.902 0.993 0.024
Second order qt = k_d_q_e_^2^t /1 + k_d_q_e_t	k_d_ = apparent desorption rate coefficient (h^−1^)	K_d_ q_e_ R^2^ SE	0.561 1000 0.849 0.084	0.361 714.3 0.717 0.168	0.875 1666 0.913 0.075	0.028 2000 0.955 0.014	0.011 1000 0.787 0.066	31.62 11,111 0.472 0.032

C = Classical fertilizers (control) ^a^ SE|≤ 0.101 in all the parameters

**Table 3:** Kinetics models for Mg release from classical fertilizers (control) and NEF in water and soil

Models release kinetics	Description	Parameter	Water			Soil		
			nDPF1	nDPF2	**C**	nDPF1	nDPF2	**C**
First order Ln(q_0_–q_t_) = a − (K_d_ * T)	q_0_ = amount of Mg released at equilibrium mg·g^−1^, q or q_t_ = amount of Mg released at time t (h) mg·g^−1^, K_d_ = Solubility rate (h^−1^) a = constant (mg.g^−1^)	K_d_ a R^2^ SE	0.012 5.657 0.979 0.112	0.011 5.302 0.929 0.210	0.012 5.967 0.975 0.129	0.006 3.215 0.911 0.221	0.006 2.753 0.913 0.261	0.006 3.178 0.911 0.207
Elovich q_t_ = (1/β)ln(αβ) + (1/β)lnt	α = initial desorption rate of Mg (mg/g·h) β = constant related to Mg release (mg.g^−1^)	α β R^2^ SE	63.92 0.021 0.875 38.29	3E + 01 0.032 0.837 29.78	5E + 01 0.015 0.882 50.58	0.1506 0.140 0.898 1.820	8E-02 0.221 0.916 1.035	2E-01 0.142 0.882 1.945
Parabolic diffusion q = a + k_d_t^1/2^	a = constant (mg.g^−1^) K_d_ = apparent diffusion rate coefficient (mg/g.h^1/2^)	K_d_ a R^2^ SE	20.41 22.87 0.986 11.55	13.43 4.807 0.964 12.47	27.95 6.495 0.993 11.47	1.199 7.378 0.971 0.982	0.753 5.514 0.974 0.579	1.183 6.576 0.961 1.127
Power function^a^ logq = logk_d_C_ο_ + 1/m logt	K_d_ = apparent desorption rate coefficient (h^−1^) 1/m = constant Co = initial Mg concentration	K_d_ 1/m R^2^ SE	44.96 0.349 0.977 0.047	23.65 0.379 0.962 0.067	29.70 0.478 0.992 0.035	0.050 0.959 0.994 0.024	0.002 1.416 0.988 0.050	0.110 0.832 0.985 0.034
Second order q_t_ = k_d_q_e_^2^t /1 + k_d_q_e_t	k_d_ = apparent desorption rate coefficient (h^−1^)	K_d_ q_e_ R^2^ SE	0.518 333.3 0.917 0.059	0.317 222.2 0.849 0.121	0.438 434.8 0.899 0.051	0.013 1250 0.070 0.364	0.014 81.30 0.598 1.282	0.027 500 0.334 0.365

C = Classical fertilizers (control) ^a^SE|≤ 0.1 in all the parameters

In conclusion, this study demonstrates the potential of nano-enabled fertilizers from green waste for sustainable and controlled release of nutrients. The use of green waste as a source of nutrients not only reduces waste but also provides an environmentally friendly alternative to traditional chemical fertilizers. Further studies are needed to optimize the preparation of nano-enabled fertilizers and to investigate their long-term effects on crop growth and yield.

### Plant growth parameters

**Fig. 11:**
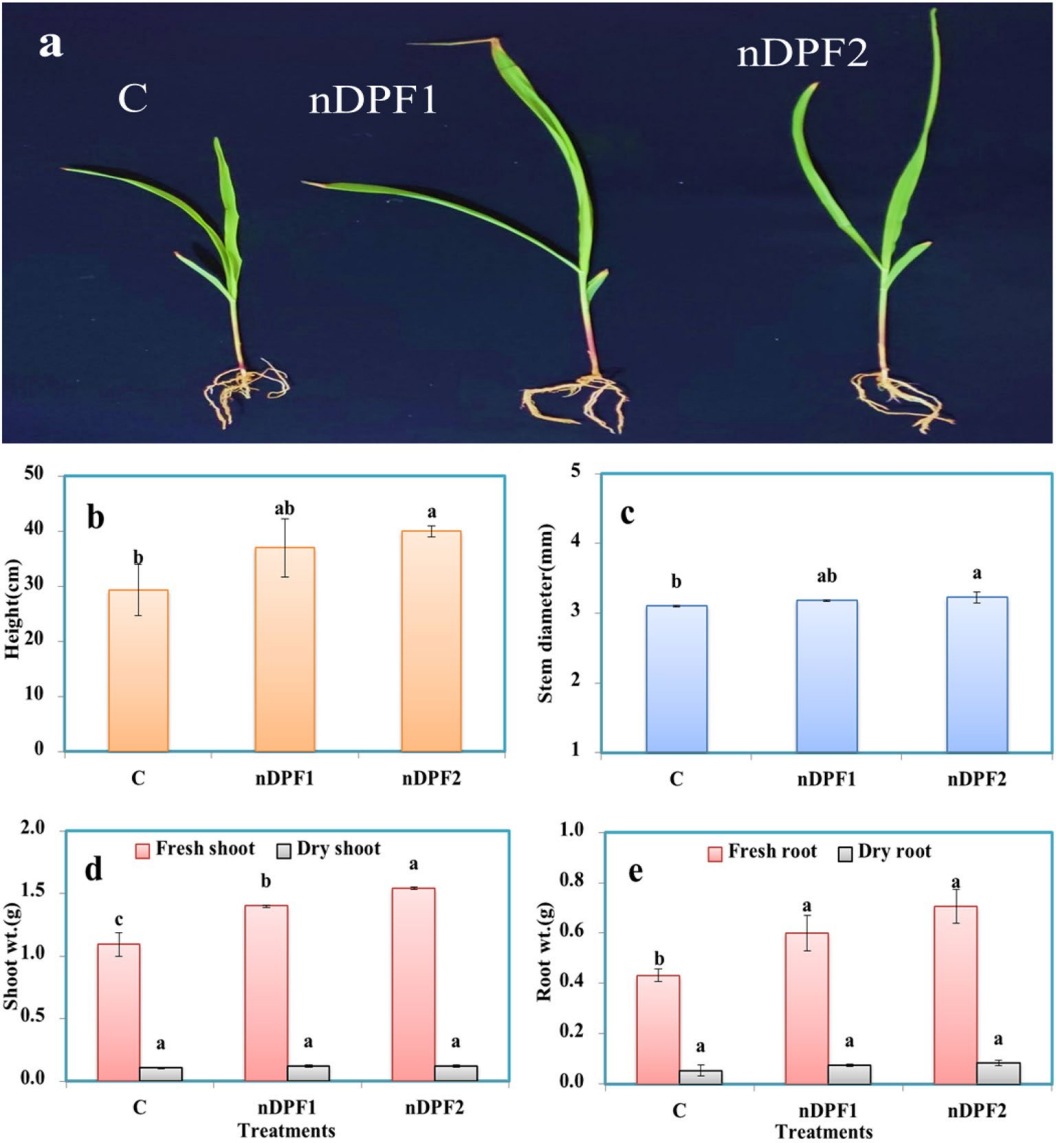
Picture of corn (Zea maize L.) plants at 25 days (**a**) plant height (**b**) stem diameter (**c**) shoot (fresh and dry) weight (**d**) root (fresh and dry) weight (**e**)

**Fig. 12:**
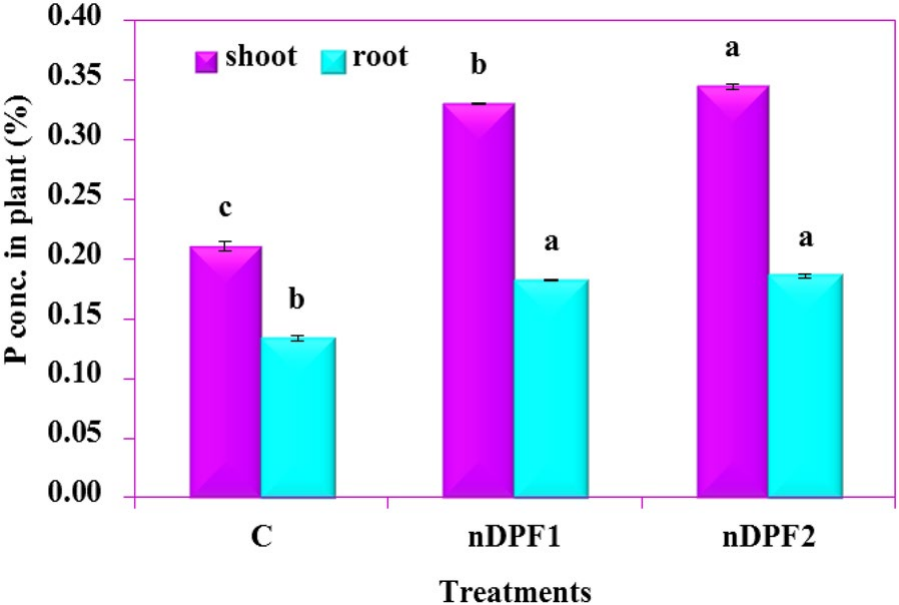
Phosphorus concentration in plant tissues

The ENF fertilizers showed a positive influence on early stage of seedling growth (Fig. 11). The data presented revealed significant influences of the tested nano-enabled fertilizers (nDPF1and nDPF2) on plant growth as compared to that of commercial fertilizer (control). Both nDPF1and nDPF2 treatments gave the highest values of plant height (37 cm and 40 cm), respectively, whereas the lowest value (29.27 cm) was obtained from the commercial fertilizers treatments (control) (Fig. 11b). Similarly, stem diameter, shoot fresh and dry weights and root fresh weight were significantly higher those of the commercial P fertilizer treated soil (Fig. 11c, d, e). Phosphorus content in plant shoot and root in nDPF2 treatment achieved the highest values of P% (0.35% in shoot and 0.186% in root) whereas the nDPF1 treatment showed an intermediate effect on P content in plant tissues (0.33% in shoot and 0.182% in root). A significantly lowest P content in plant tissues (0.21 in shoot % and 0.13% in root) was detected in the control treatment plants (Fig. 12). The significant increase of P content in plants treated with nDPF1 and nDPF2 was beneficial for the plants, leading to improvement of maize growth (Mandal et al. 2009). Because the use of nano-enabled fertilizer can retain a large quantity of nutrients for gradual and consistent release, the significant increase in P content in plants treated with nDPF1 and nDPF2 was beneficial for the plants, leading to improvement in growth** (**Mandal et al. 2009; Ghormade et al. 2011**)**. Therefore, the enabled nano-fertilizers have the potential to increase efficient delivery of nutrients to plants, reduce the loss of nutrients through leaching.

## Conclusions

Promising nano-enabled fertilizers (nDPF1 and nDPF2) were produced by impregnation of nanostructured date palm pits (nDPP) with (KH_2_PO_4_ + MgO) at 1:1 and 3:1 (w/w) ratios respectively using a planetary ball mill. Application of the produced nano-fertilizers to the sandy soil effectively improved water retention capacity of the soil during the 60days study with nDPF2 being the most efficient. The water content of the control soil was almost evaporated after 26 days, whereas the soil treated with nDPF2 fertilizer showed water retention value of 11% after 60days. The leaching behavior study demonstrated that nDPF2 significantly controlled P–K–Mg nutrients release due to its lowest leaching losses of P–K–Mg (8.9–2.9–16.9%) respectively in comparison with nDPF1 (10.8–6.3–26.1%) and classical fertilizer (22.4–35.7–47%). These results showed that the nano-enabled fertilizers exhibited a sustained release of nutrients, with a gradual decline in the release rate over time. This is a significant advantage over traditional fertilizers, which release nutrients quickly and can lead to leaching and nutrients losses. The low standard error (SE) values of power function model verified its high potentiality to predict NEF release data and ascertained that the reversibly P, K and Mg adsorbed phases were mainly related to the initial concentration of P, K and Mg and proportional to the fractional power of time. The pilot experiment was carried out to evaluate the effect of the proposed NEF on growth and P content of maize plants within a short time period of 25 days after fertilizers application. The Pot study demonstrated that the use of enabled nano-fertilizers may help to maximize nutrients use efficiency and minimize nutrient loss by leaching as well as industrial agricultural waste. However, long term future studies at field scale should be conducted to validate these data and to assess plant productivity, nutrients availability together with their environmental impacts.

### Supplementary Information

**Additional file 1: Table S1.** The physical and chemical characterization of the tested soil. **Table S2.** XPS analysis of nDPF. **Fig.S1.** Zeta potential of nDPP (A) and SEM image of MgONPs (B).

## Data Availability

The datasets used and/or analyzed during the current study can be available from the corresponding author upon reasonable request.

## Abbreviations

nDPP: Nanostructured date palm pits
TEM: Transmission electron microscopy
XRD: X-ray diffraction analysis
XPS: X-ray photoelectron spectroscopy
SEM: Scanning electron microscopy
EDX: Energy dispersive X-ray
NEF: Nano- enabled fertilizers
FTIR: Fourier transform infrared spectroscopy
