# Supplementary material for: Eco-friendly nano-enabled fertilizers derived from date industry waste for sustainable and controlled-release of P, K and Mg nutrients: sorption mechanisms, controlled-release performance and kinetics
Source: Bioresour Bioprocess. 2024 Jan 3;11(1):3. doi: 10.1186/s40643-023-00716-6 (PMC10991569; doi:10.1186/s40643-023-00716-6)
Supplement: Supplementary file 1 — Additional file 1: Table S1. The physical and chemical characterization of the tested soil. Table S2. XPS analysis of nDPF. Fig.S1. Zeta potential of nDPP (A) and SEM image of MgONPs (B). [file 40643_2023_716_MOESM1_ESM.docx]

**Supplemental Materials**

**Soil Location and sampling**

Soil samples were collected from the rear of the 0-30 cm from EL- Alamien, Alexandria, Egypt, and transported into the laboratory.

**Soil characteristics**

The chemical and physical characteristics of the soil used in controlled release studies are presented in Table (S1). The soil was sandy, moderately alkaline (pH 8.2) with low OC, EC and low nutrients content.

**Table (S1)** The physical and chemical characterization of the tested soil

| **Soil parameter** | **Sandy soil** |
| --- | --- |
| pH | 8.20 |
| EC (dS/m) | 0.61 |
| Total N (%) | 0.003 |
| Available P (mg/kg) | 5.34 |
| Organic C (%) | 0.044 |
| Sand (%) | 92.96 |
| Silt (%) | 0.21 |
| Clay (%) | 6.83 |
| Texture | Sandy |
| **Soluble ions (mg/kg)** |  |
| Ca^2+^ | 51.60 |
| Mg^2+^ | 11.76 |
| Na^+^ | 55.20 |
| K^+^ | 3.90 |
| CO_3_^2-^ | 0.00 |
| HCO_3_^-^ | 109.80 |
| SO_4_^-^  Cl^-^ | 12.48  142.00 |

D. L. Sparks etal.2001, Methods of Soil Analysis , Part 3: Chemical Methods. Soil Soil Science Society of America, Inc.Madison, Wisconsin, USA.

**TEM sample preparation techniques**

The pits powder was dispersed in ethanol and sonicated for 15 min, 5 µl of the dispersed solution was dropped on a carbon coated copper grid then lefted until complete drying at room temperature. The tem characterization was done at 200 kV. The particle size was measured using Gatan digital micrograph software.(JEOL.JEM-2100F).

**SEM sample preparation techniques**

The sample powder was stacked into double faced carbon tab and then coated with

Pt-Pd coating source using JEOL auto fine counter, JEC- 560 ( JEOL JSM 6010-LV)

**XRD methodology**

Crystal structure of nDPP powder was performed via X-ray diffraction analysis. X-ray diffractogram using a broker Meas Srv (D2-208219)**/** D2-208219 diffractometer that operates at 30 Kv, 10 Ma with Cu tube ( λ = 1.54 A˚),with a range from 2 ^◦^ to 100 ^◦^

**Water-retention behavior of nDPF1 and nDPF2 in sandy soil**

The dried sample (1g) of the nano-enabled fertilizer (NEF) was thoroughly mixed with 100 g of dried sandy soil in a 250 mL of beaker. Meanwhile, a control of 100 g of dried sandy soil alone was placed in separate beaker. A 50 mL of distilled water was then added to each beaker and weighed. The weight of the beakers was recorded every three days at room temperature until constant mass is reached. The soil water-retention WR (%) was calculated using Eq. (1):

W_r_= W_t_−W/W_0_−W X100% (1)

Where W_r_ is water-retention rate and W is the total mass of the used soil and beaker. W_0_ is the total mass of sand, beaker, and distilled water added. W_t_ is the total mass of sand, NF and beakers at regular intervals (Gungula et al., 2021).

***Slow release behavior of nDPF1 and nDPF2***

The release pattern of nDPF1 and nDPF2 were first investigated in distilled water. Briefly, 0.1 g of dried NEF sample and 10 mL of distilled water were added into a dialysis bag, and then placed in an Erlenmeyer flask containing 200 mL of distilled water. A 10 mL of the solution was then withdrawn from the flask at preset time intervals and 10 mL of fresh distilled water was added to maintain constant volume. The P, K and Mg content in the solution were determined using ultraviolet spectrophotometry/flame photometry. Moreover, the release behavior of nDPF1 and nDPF2 in soil were also studied. Briefly, PVC columns with 8 cm diameter of 30 cm height and filter paper with 200-mesh-nylon-cloth–sealed bottom were used. A mixture of 1 g dried NEF, 300 g dried sandy soil was placed in the column and 70 mL of distilled water was added to each soil column every 48 hrs. At preset intervals, 10 mL of soil leaching solution was collected and the P, K & Mg contents in the aqueous solution were determined. The amounts of P, K & Mg released per g of fertilizers [q (mg/g)] in water and soil were calculated using the following equation:

q =Ci*V/mb (2)

Where Ci is the P, K& Mg concentration (mg/L), V is the volume of water (L), and mb (g) is the mass of the nDPF1 and nDPF2and control added in to water (Qian et al., 2013).

The cumulative release CR (%) were calculated by Eq.(3).

CR =V_E_∑^n−1^C_i_ +V_0_Cn/M_0_ X 100% (3)

Where CR is the cumulative release rate, V_E_ and V_0_ are the initial volume of the sample volume and release medium, respectively. C_i_ and C_n_ are the fertilizer concentrations, i and n are the sampling times, M_0_ is the total mass of the fertilizer loaded in the sample (Wei et al., 2019).


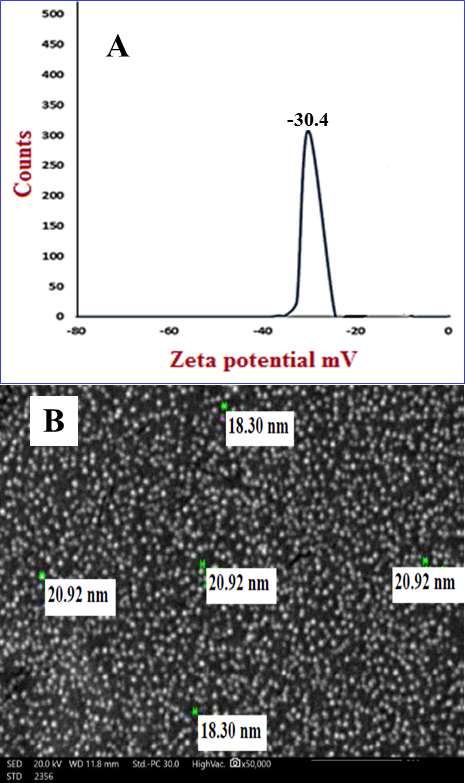


**Fig.S1.** Zeta potential of nDPP (A) and SEM image of MgONPs (B)

**Table S2.** XPS analysis of nDPF

| Name | Peak BE | FWHM eV | Area (P) CPS.eV | Atomic % |
| --- | --- | --- | --- | --- |
| O1s | 533.44 | 4.45 | 240985.8 | 64.17 |
| Si2p | 105.12 | 4.76 | 27085.26 | 21.33 |
